# A hand-held, power-free microfluidic device for monodisperse droplet generation

**DOI:** 10.1016/j.mex.2018.08.008

**Published:** 2018-08-20

**Authors:** I-Jane Chen, Tao Wu, Shuhuan Hu

**Affiliations:** a Complete Genomics Inc., San Jose, CA, USA; b Beijing Genomic Institute, Shenzhen, Guandong, China

**Keywords:** Droplet generation, Emulsion, Monodisperse, Single cell sequencing, Digital PCR, Portable device

## Abstract

Here we develop a microfluidic device to generate monodispersion sub-nanoliter size droplets. Our system reaches steady state within 3 s after the flow starts and generates 100,000 droplets in 28 s with high size consistency (CV < 8%). This low cost device is composed with a microfluidic chip, 2 tubings, a collection vial, a syringe and a station; and is in the size of an iPad Mini (4” × 6” × 3/4”). In this system, all incoming reagents share the same pressure drop across the fluidic passage to generator droplets. A single source negative pressure is applied to the fluids to create the flow by a vacuum at the exit end of the device. The vacuum is generated on-site by pulling the plunger of a syringe. The position of the plunger before and after pulling determines the degree of vacuum. A fixture is used to hold the plunger after it is pulled to maintain its vacuum. Although this system loses vacuum gradually as the liquid filling in, it maintains a flow rates with the changes less than 10% and droplet sizes changes less than 2% during the course of generating 150,000 droplets. The pressure drop across the chip, the flow rates of all reagents, the droplet size and generation frequency are predictable, programmable, and reproducible. This device is designed for generating droplets for single cell genome profiling application but can be also used for digital PCR or other droplet-based applications.

## Graphical abstract

**Figure:**
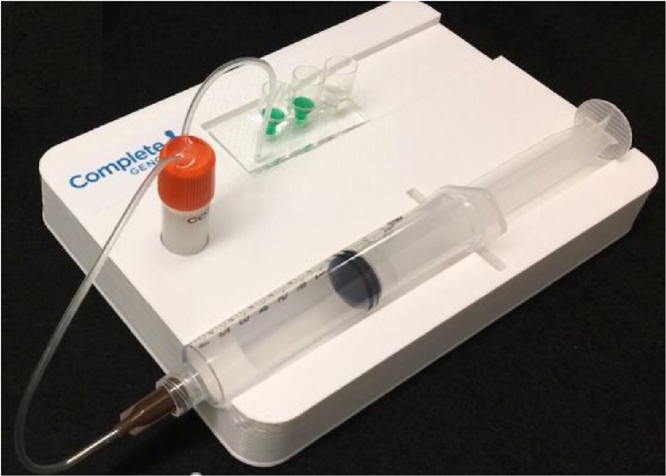

##   

**Specifications Table**

**Table:** 

Subject area	*Engineering*
More specific subject area	*Microfluidic Device*
Method name	*Monodisperse droplet generation using a passive setup*
Name and reference of original method	1. *Droplet generation using 3 individual pumps, Highly Parallel Genome-wide Expression Profilingof Individual Cells Using Nanoliter Droplets, Macosko EZ, et. al., Cell. 2015 May 21;161(5):1202-1214*. 2. *Droplet generation using 1 pump, W Stephenson, et. al., NATURE COMMUNICATIONS (2018) 9:791*.
Resource availability	*ImageJ, an open source image processing software*. https://imagej.nih.gov/ij/

## Background

Droplet-based technologies catch a lot of interests during the past decades for its capability of screening very large numbers of samples simultaneously (usually tens of thousands). By dividing the sample and reaction reagents into separate small volume reactors, each sample is expressed individually. Such kind of platform can be used to quantify and qualify cellular genomic information at individual cell level (such as digital PCR, single cells genome profiling).

In a droplet-based system, two or more reagents are loaded in the reservoirs at the inlet of this device. The reservoirs are connected via microfluidic channels which intercept at least once at junction(s). An exit channel connects the junction and the exit port and allows fluid to exit the device. As the fluids traveling though the channels and intercept at the junction, continuous phase reagent pinches the discret phase reagent either by focusing flow (a cross-junction setting) or concurrent flow (a T-junction setting). The balance between the inertia of discret phase solution, surface tension between discret and continuous phase and viscosity enable continuous phase reagent to encapsulate the discret phase reagent and form droplets of discret phase solution. The dimension of the droplets are determined by the flow rates of all reagents (Q), surface tension (σ) between these two immiscible phases, viscosity (μ) of the continuous phase, density (ρ) of the continuous phase, the channel height (h), channel hydraulic diameter (d) at the junction and the channel width (w) at the exit of the junction are the junction. The correlation is expressed as below in Eqs. (1a) and (1b) [1]

(1a) $$R_{droplet\;}=\;\frac{3.7\mu_{o}w_{down}h}{4\rho_{o}Q_{o}}e^{\frac{-4}{3w_{down}}[\frac{6\mu_{o}Q_{o}\pi^{2}d_{j}^{2}}{\pi^{2}d_{j}^{3}\sigma -4\rho_{w}Q_{w}^{2}}+\beta h]},\;{\;\;R}_{droplet}>h/2$$

(1b) $$R_{droplet\;}=\frac{w_{down}h(\pi^{2}d_{j}^{3}\sigma -4\rho_{w}Q_{w}^{2})}{{6\mu }_{o}Q_{o}\pi^{2}d_{j}^{2}+2\beta h(\pi^{2}d_{j}^{3}\sigma -4\rho_{w}Q_{w}^{2})},{\;\;R}_{droplet}<h/2$$

**Fig. 1:**
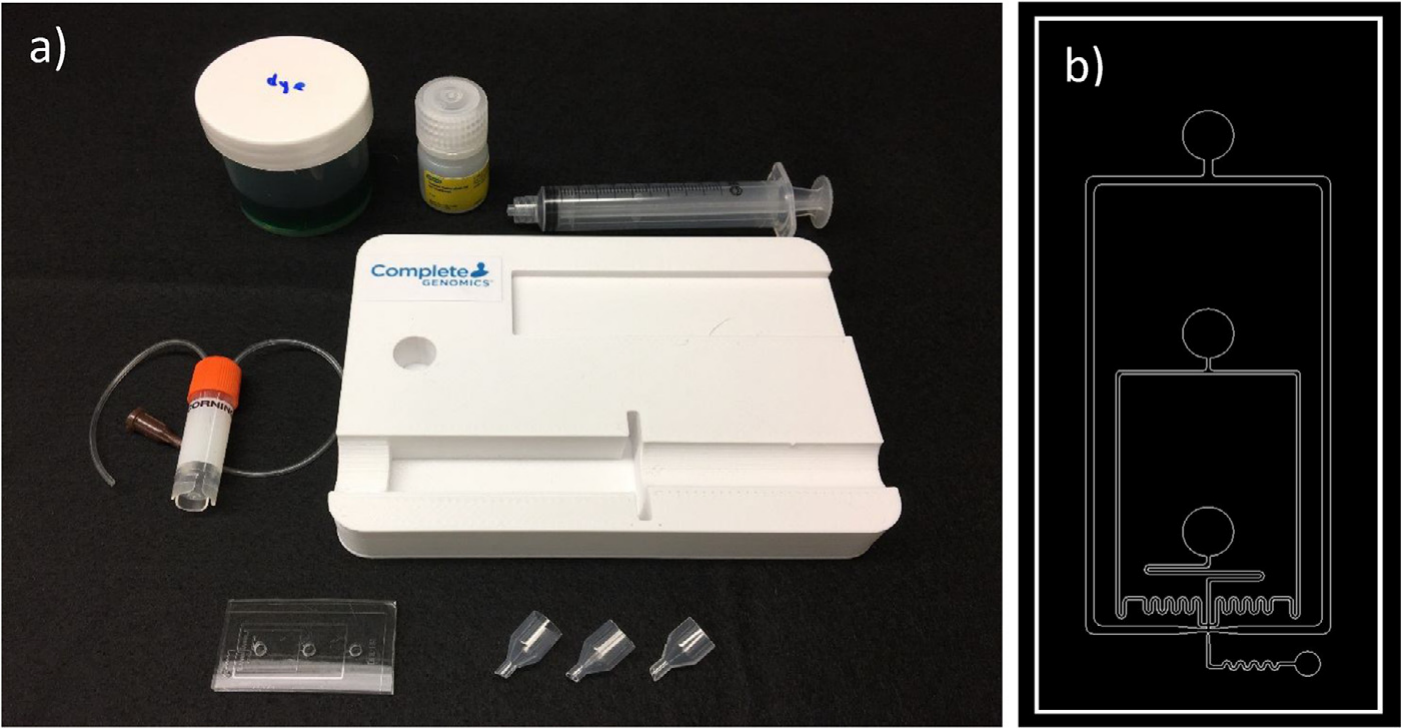
System setting and the design of the droplet generation chip. a) The system is composed with a PDMS microfluidic chip, 3 external reservoirs, a droplet collection vial with 2 tubings, a syringe and a station. b) From the inner-most to the outer-most port of the chip accommodates beads, cells, and droplet forming oil. The lower right port is connected to the syringe via collection tubing.

In this research, we adopted the parameters of generating nanoliter size droplets by keeping the flow rate of 3 liquids at 5, 5 and 10 mL/h (water/water/oil) [2]. However, instead of using three individual syringe pumps to drive the liquid, we develop a microfluidic device, of which all three incoming reagents share the same pressure drop (ΔP) through the fluidic passage. The channel length, width and height and liquid viscosity determine the resistance (R) of each channel, as showed in the equations below in Eq. (2). The channel height of this device is 120 μm. The system therefore generate flows at the flow rate ratio at 1:1:2. The design of the chip is showed in Fig. 1a. The AutoCAD file is available upon request.

(2) $$\begin{array}{lll} \text{Q}=\text{ΔP/R}; & \text{ΔP}={\text{P}}_{\text{atm}}-{\text{P}}_{\text{syringe}}; & R_{rec}=\frac{12}{\left(w/h\right)-0.63} \end{array}\frac{\eta L}{h^{4}}\;\text{for}\;w>h$$

A single source negative pressure is applied to the fluids to create the flow by a vacuum at the exit end of the device. The vacuum is generated on-site by pulling the plunger of a syringe. The positions of the plunger before and after pulling determine the degree of vacuum following ideal gas law P_initial_V_initial_ = P_final_V_final_. A fixture is used to hold the plunger at the “pulled position” to maintain its vacuum. This microfluidic chip is designed to generate 20 mL/h flow rate when the pressure drop is 1.73 × 10^4^ Pa. As the liquids fill in and droplets are generated, the air in the syringe is compressed and vacuum is lost gradually. An analysis based on Eqs. (1a) and (1b) predicts when the change of pressure drop (ΔP) is smaller than 10%, the change of flow rate is therefore below 10% and the change of droplet size is not greater than 2%. The pressure drop across the chip, the flow rates of all reagents, the droplet size and generation frequency are therefore predictable, programmable, and reproducible.

This low cost device is composed with a microfluidic chip, a tube, a collection vial, a syringe and a station; and is in the size of an iPad Mini. On the contrary to commercial instruments that utilize multiple pumps yet still facing challenges of fluctuating flow rate and flow rate ratio, this invention is 100% portable and power-free. Our device can be used to generate various dual phase droplets systems such as of water-in-oil, oil-in-water, water-in-water (emulsion), oil-in-oil system; or more complex, multi-layer systems, such as water-in-oil-in-water, etc. Its application includes, but not limited to, generating droplets for high throughput, parallel droplet-based mRNA or DNA profiling, digital PCR, and emulsions for pharmaceutical applications.

## Method details

### Materials

Tygon Microbore Autoanalysis Tubing, OD 1/16”, ID 1/50”, CP ND-100-80 Cole-Parmer, USA. Droplet Generation Oil for EvaGreen, # 1864005, Bio-rad, USA. Disposable Biopsy Punch, 1.5 mm and 3 mm, # 33-31 A, # 33-32, Miltex, USA. PDMS, Sylgard 184, Dow Corning, USA. Glass slide, 75 × 25 mm, 48300-026, VWR, USA. External Thread Cryogenic vial, 2.0 mL, self-standing, # 430659, Corning, USA. Ten milliliter syringe, # 302995, BD, USA. 19 G dispensing needle (blunt needle is strongly recommended), # 901-19-050, CMLsupply, USA. Mylar mask, Shenzhen Qingyi Photomask, China. SU8 mold, Suzhou Chip Scientific Instrument, China. 2-parts Epoxy, Devcon, USA. Plasma Cleaner, PDC-001, Harrick Plasma, USA. Plastic dropper, EW-06226-30, Cole Parmer, USA. Dish soap, Dawn Ultra, P&G Group, USA. Silicone glue, clear Silicone II, GE, USA.

### Protocol

A Microfluidic Chip Fabrication
  1 Microstructure mold is made with SU-8 on a 4”-silicon wafer at the height of 120 μm by photolithography method. The mold needs to be silanized prior use.
  2 Leave the mold in a 5” Petri dish with the micro-features facing up.
  3 Mix fifty grams of PDMS (curing agent and polymer base at the ratio of 2:10) thoroughly (3 min). Pour forty grams of the mixture on the SU-8 microstructure mold and pour the remaining on another 4” Petri dish. Leave the Petri dishes in a desiccator and vacuum for 5 min until all air bubbles disappear in PDMS mixture.
  4 Bring the Petri dishes to an oven and let solidify at 70 °C for 30 min.
  5 Score the cured PDMS along the circumference to separate from the Petri dish. Peal the PDMS off from the mold. With the featured side facing up, use hole punch to make connection ports. (inlet ports with a 3 mm hole punch and outlet port with a 1.5 mm punch). Make sure the featured PDMS is at least 3-mm thick. Double check the holes are totally clear without any PDMS stuck in. Note: Punching PDMS too rapidly will result in cracks and future leakage. It is recommended to practice on dummy PDMS until holes come out nicely.
  6 PDMS specimens are washed in diluted dish detergent (1:100) using a sonicator for 10 min, followed by replacing the soapy water with DI water and sonicated twice. Make sure the specimens are totally immersed and never overlap during washing. When handling PDMS, wear powder-free gloves.
  7 Blow dry the PDMS specimens using purified, compressed air.
  8 Place PDMS in plasma chamber. Keep featured-side facing up. Vacuum the chamber down to 1 torr then activate plasma for 1 min.
  9 Bring out the PDMS specimens and carefully place the 3-mm PDMS with features facing down on top of the flat PDMS.
  10 Incubate the PDMS at 70 °C for 3 h for bonding.
B Collection vial
  1 Use 3 mm hole punch to punch 2 holes on the silicone septum of the sample collection vial.
  2 Insert two 15-cm long Tygon tubings (A and B) through the holes. Adjust the tubings so tubing A has about 1 cm inside of the vial and tubing B touches the bottom of the vial. Apply epoxy as additional seal if necessary.
  3 Load 100 μL Droplet generating oil in sample collection vial.
  4 Screw the cap on the vial. Make sure the Droplet generating oil does not leak out.
  5 Insert a 19 G blunt needle at outer end of the tubing A. Connect the needle with a 10 mL syringe.
C System Setup
  1 The station is made with PLA by 3D printing. The CAD file is available upon request.
  2 Cut off bulb part of 3 plastic droppers with remaining height of 5 mm as reservoirs, cut off the straw part of the droppers with remaining length of 2 mm as inserts for PDMS chip as showed in Fig. 1. Make sure the inserts are cut with an angle close to but not exactly at 90 degrees angle to prevent flow blockage. Note: 200 μL pipette tips can be used in the replacement of plastic dropper. It is, however, not recommended since pipette tips tend to trap air bubbles and interfere with flow.
  3 Insert all reservoirs to inlet ports and tubing B to outlet port. Note: Users can apply a thin layer of silicone glue at the reservoir insert and the tip of tubing to assure all junctions are air-tight. To verify if the system is air-tight, load 100 μL water in all three reservoirs and pull plunger to 10 mL tick quickly and then release. Water should not leak out from the junction and the plunger should re-track to its initial position within a few seconds.
  4 Pull syringe plunger to 7.0 mL tick.
  5 Load 100 μL aqueous solution A at reservoir 1.
  6 Load 100 μL aqueous solution B at reservoir 2.
  7 Load 150 μL droplet generating oil at reservoir 3. Note: To minimize droplet size variation, please follow the sequences step 6 through 8.
  8 Pull plunger to 9.0 mL tick quickly and rest the syringe on the station with plunger held in position by the fixture.
  9 Unscrew the sample vial cap 40 s after flow starts. Breaking the vacuum will stop the flow. Extending the flow time will result in inconsistent droplet sizes. Note: To minimize droplet size variation, please make sure to follow this step.
  10 Collect the droplets from the top layer of the collection vial (droplet generation oil is heavier than droplets). About 150,000 droplets are generated within this timeframe.

## Method validation

**Fig. 2:**
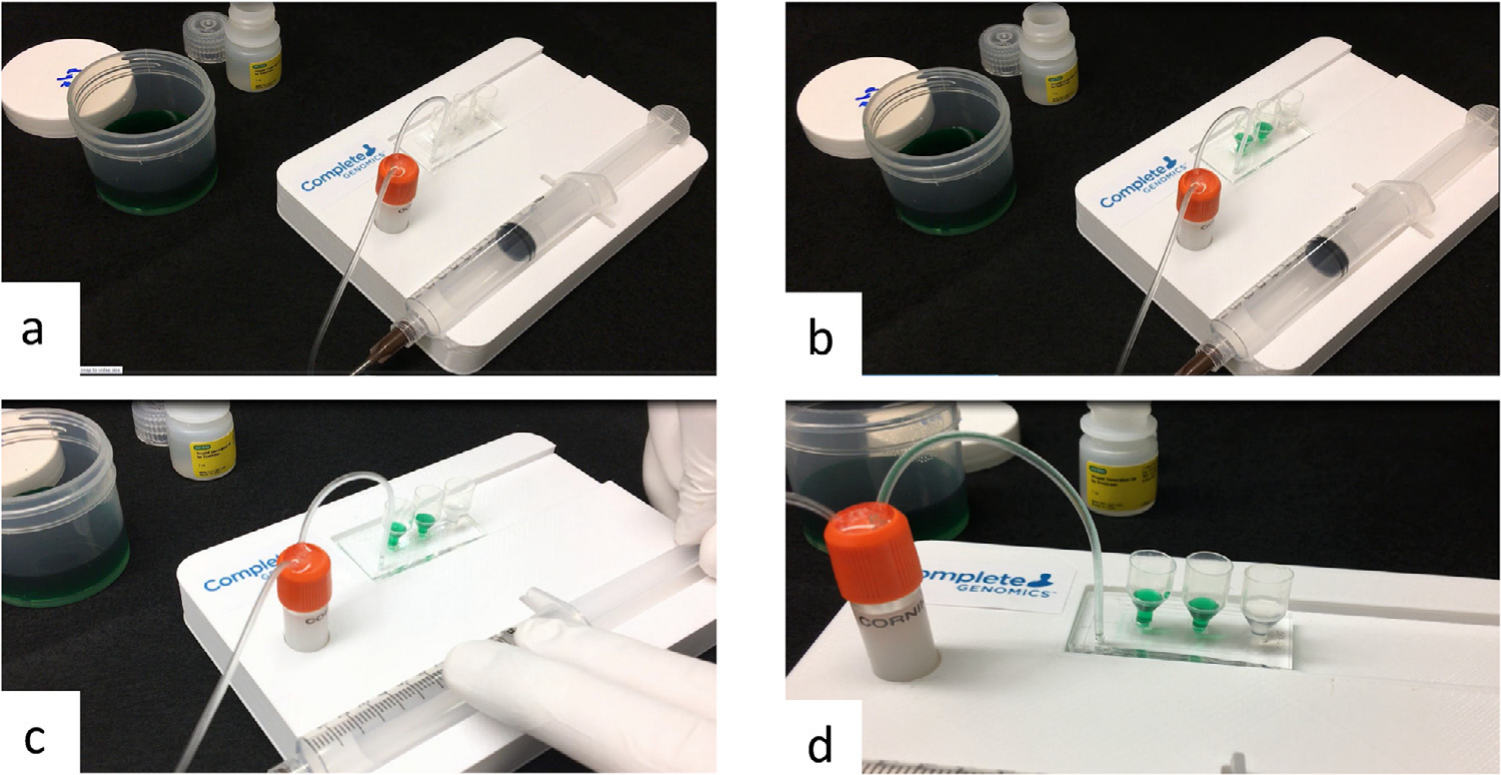
Step-by-step workflow. a) Set up the system. Pull plunger to 7.0 mL tick. b) Load aqueous and oil solutions to the reservoirs. c) Pull the plunger to 9.0 mL tick quickly and rest the syringe on the station. d) Droplets are formed in the chip and flow to collection vial.

**Fig. 3:**
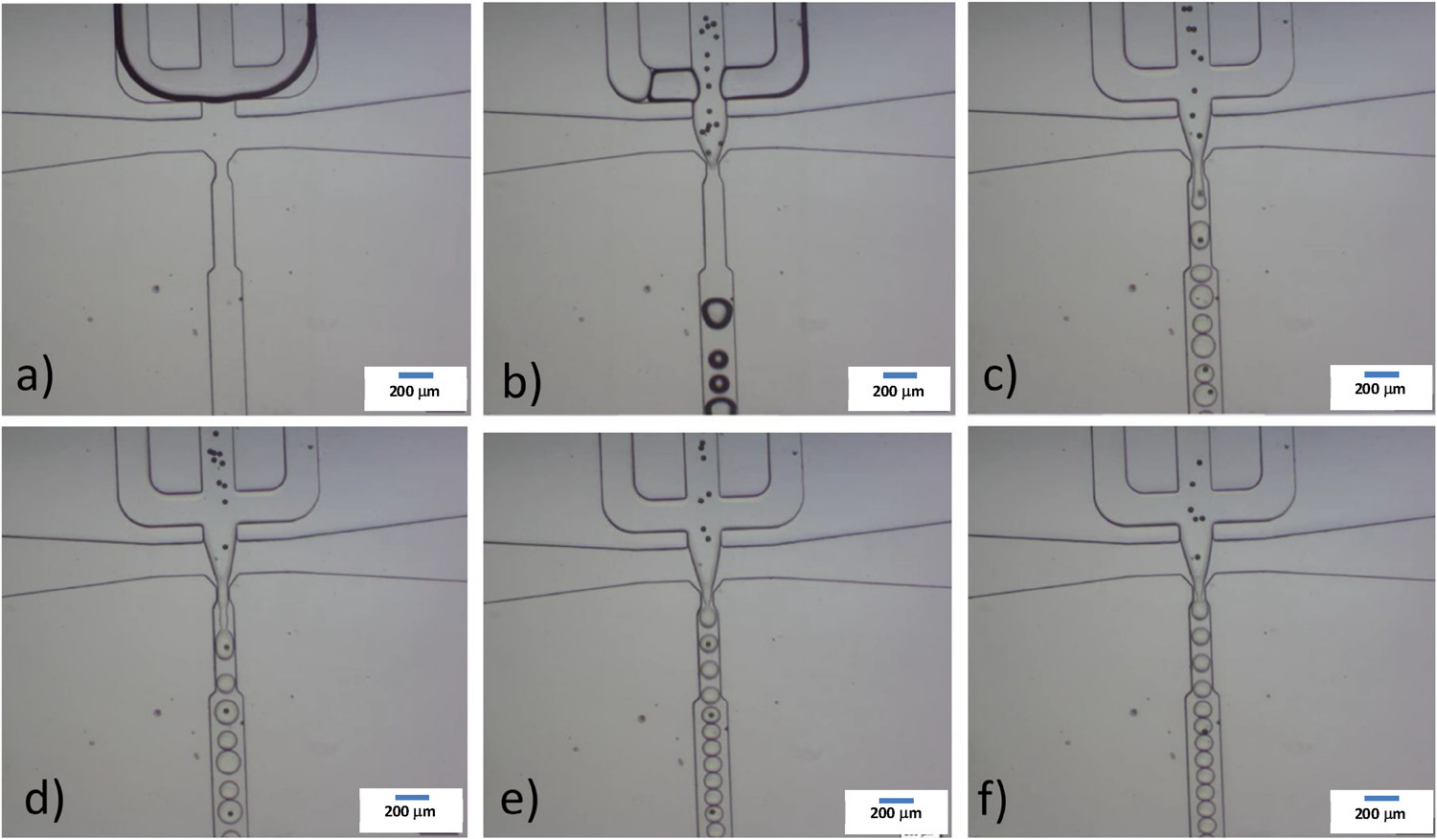
Droplet formation. Droplets composed with an aqueous solution with magnetic beads and another aqueous solution are formed at the junction. Real-time close-up images during the transit and steady state are showed at time a) 0 s; b) 1 s; c) 2 s; d) 3 s; e) 4 s; and f) 25 s after flow is actuated. The scale bar represents a length of 200 μm.

**Fig. 4:**
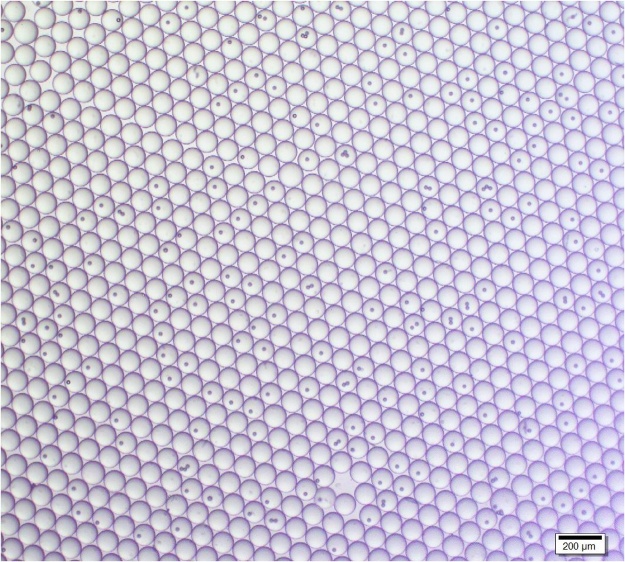
Droplets displayed on a cover slip. Twenty microliter of solution collected in the collection vial is placed on a glass slide and shows droplets with high size consistency (109 μm ± 9 μm).

The system was validated by two settings. First setting is designed to encapsulate aqueous solution mixed with green food grade dye for better visualization; second setting is designed to encapsulate magnetic beads (30 μm, 350 bead/μL) for single cell sequencing feasibility test. (Beads and cells have higher density than water and tend to sediment in the reservoirs and result in flow blockage. We have developed a method to mitigate this issue and will discuss in future publication) Fig. 2 shows that the droplets being formed in the chip and flow throw the tubing into the collection vial. Fig. 3 shows time-lapse images of droplet formation from the beginning of the flow untill 28 s after flow started, with 100,000 droplets generated. Fig. 4 shows solution in the collection vial contains droplets with high size consistency. 20-μL aliquots from multiple locations of the collection vial were acquired, spread on a glass slide and imaged right away. The droplet size was analyzed using ImageJ (an open source software). The average size of the droplets is 109 μm, with standard deviation of 9 μm (CV < 8%).
